# Cervical cancer in Malaysia: can we improve our screening and preventive practice?

**DOI:** 10.1186/1471-2458-12-S2-A17

**Published:** 2012-11-27

**Authors:** Shanthi Varatharajan, M Majdah, Syed Aljunid, Won-Sun Chen, A Mukarramah, Chee-Meng Yong

**Affiliations:** 1 Clinical Research Centre, Ministry of Health Malaysia, Jalan Pahang, 50586 Kuala Lumpur, Malaysia; 2 Family Health Development Division, Department of Public Health, Ministry of Health Malaysia, 62518 Putrajaya, Malaysia; 3 Department of Community Health, Universiti Kebangsaan Malaysia Medical Centre, Jalan Yaacob Latif, 56000 Kuala Lumpur, Malaysia; 4 United Nations University- International Institute of Global Health, Universiti Kebangsaan Malaysia Medical Centre, Jalan Yaacob Latif, 56000 Kuala Lumpur, Malaysia; 5 Research & Innovation Management Centre (RIMC), SEGI University College, Jalan Teknologi, 47810 Petaling Jaya, Malaysia; 6 Pathology Department, Hospital Raja Perempuan Zainab II, Ministry of Health Malaysia, 15586 Kota Bharu, Malaysia; 7 Gynecology Department, Hospital Ampang, Ministry of Health Malaysia, 68000 Ampang , Malaysia

## Background

The 2010 WHO/ICO (Institut Catala d’Oncologia) summary report states that Malaysia has a population of 8.7 million women aged 15 years and above who are at risk of developing cervical cancer. Annually, 2126 women are diagnosed with cervical cancer and 631 die from the disease. The introduction of HPV vaccine in Malaysia has significantly encouraged the introduction of molecular era which could potentially improve the future of cervical cancer screening program. This article aimed at reviewing the proposed methods published from different perspectives in improving the screening and preventive practices of cervical cancer in Malaysia.

## Materials and methods

Published articles and reports from year 2000 onwards were retrieved from electronic and government databases. Selected studies include randomized controlled trials (RCTs), non-randomized controlled trials (CCTs), cross sectional studies and cost-effectiveness studies. Selected interventions were accepted models for improving cervical cancer screening program. Selected outcomes had at least one of following: reduced morbidity/mortality of cervical cancer, increased detection of precancerous/cancer lesions, increased coverage of screening, increased awareness and/or knowledge and/or barriers to cervical cancer, HPV and vaccination. Search terms included cervical cancer, screening, HPV, vaccine, Malaysia, diagnosis, pathology, pap smear, HPV DNA test and early detection. Articles were reviewed by two reviewers, for research design and for grading internal validity of study. The accepted articles were then reviewed for quality of evidence and external validity.

## Results

In the twenty-eight studies reviewed, among the challenges of cervical cancer screening (most frequently mentioned) include cost-effectiveness of vaccine, awareness and knowledge of patients’ on pap smear, human papillomavirus infection and cervical cancer. Eight of these studies were small observations on the prevalence of human papillomavirus in smears taken using new technologies like HPV DNA test and liquid based cytology. Although there was recommendation to introduce these technologies into the screening program, evidence of cost-effectiveness was unavailable.

## Conclusions

Choosing the most practical and cost-effective approach for the Malaysian setting is definitely a challenge. Evidence-based approach should be used by researchers and practitioners from various disciplines to make this a success.

